# Genetic Population Structure of Local Populations of the Endangered Saltmarsh Sesarmid Crab *Clistocoeloma sinense* in Japan

**DOI:** 10.1371/journal.pone.0084720

**Published:** 2014-01-06

**Authors:** Takeshi Yuhara, Masako Kawane, Toshio Furota

**Affiliations:** 1 Department of Environmental Science, Graduate School of Science, Toho University, Funabashi, Chiba, Japan; 2 *KYOUSEI* Science Center for Life and Nature, Nara Women’s University, Nara, Japan; 3 Faculty of Sciences, Tokyo Bay Ecosystem Research Center, Toho University, Funabashi, Chiba, Japan; College of Charleston, United States of America

## Abstract

During recent decades, over 40% of Japanese estuarine tidal flats have been lost due to coastal developments. Local populations of the saltmarsh sesarmid crab *Clistocoeloma sinense*, designated as an endangered species due to the limited suitable saltmarsh habitat available, have decreased accordingly, being now represented as small remnant populations. Several such populations in Tokyo Bay, have been recognised as representing distributional limits of the species. To clarify the genetic diversity and connectivity among local coastal populations of Japanese *Clistocoeloma sinense*, including those in Tokyo Bay, mitochondrial DNA analyses were conducted in the hope of providing fundamental information for future conservation studies and an understanding of metapopulation dynamics through larval dispersal among local populations. All of the populations sampled indicated low levels of genetic diversity, which may have resulted from recent population bottlenecks or founder events. However, the results also revealed clear genetic differentiation between two enclosed-water populations in Tokyo Bay and Ise-Mikawa Bay, suggesting the existence of a barrier to larval transport between these two water bodies. Since the maintenance of genetic connectivity is a requirement of local population stability, the preservation of extant habitats and restoration of saltmarshes along the coast of Japan may be the most effective measures for conservation of this endangered species.

## Introduction

Many estuarine ecosystems worldwide have been severely disrupted by human activities, such as overexploitation, pollution and land reclamation [1]. In Japan, more than 40% of tidal flats, including estuarine in saltmarshes, have been lost, mainly during the latter half of the 20th century [2]. The destruction of estuarine mudflats and saltmarshes by land reclamation have resulted in the reduction of local populations of many mudflat/saltmarsh-specific species [3]. For example, large-scale reclamation of tidal flats and saltmarshes along the entire coastline of Tokyo Bay, conducted during the late 1960s and early 1970s [4], resulted in the loss of most of the saltmarshes in the high intertidal zone by the end of the 1970’s. Accordingly, most mudflat/saltmarsh-specific benthic species in Tokyo Bay have since become extinct or endangered [5].

The saltmarsh sesarmid crab *Clistocoeloma sinense* Shen, 1933 is distributed throughout estuaries in subtropical and temperate eastern Asia, Japan [6], Korea [7], China [8], and Taiwan [9]. Although distributed along the Pacific coasts of central and western Honshu, Shikoku, Kyushu and Okinawa, the species is designated as an endangered species in Japan by the Japanese Association of Benthology [2] because of the very significant decrease in suitable habitat. Furthermore, Tokyo Bay, representing the northern and easternmost limits of the species geographical distribution, is separated by a distance of ca. 250 km from the nearest coastal population (in Mikawa Bay). Because the genetic structures of populations at the limits of geographical distribution are generally characterized by increased genetic isolation and differentiation [10], the conservation of such populations requires an understanding of gene flow among edge and central populations, and their connectivity through larval transport.

This investigation of local population genetic structures of the endangered saltmarsh crab *Clistocoeloma sinense* along the Japanese coast utilized mitochondrial DNA (mtDNA) analyses in order to clarify the genetic diversity and relationships/connectivity among local populations of the species. Based on the investigation, we discuss a focus on conservation strategies for the geographically isolated Tokyo Bay populations.

## Materials and Methods

### Ethics Statement

**Figure 1:**
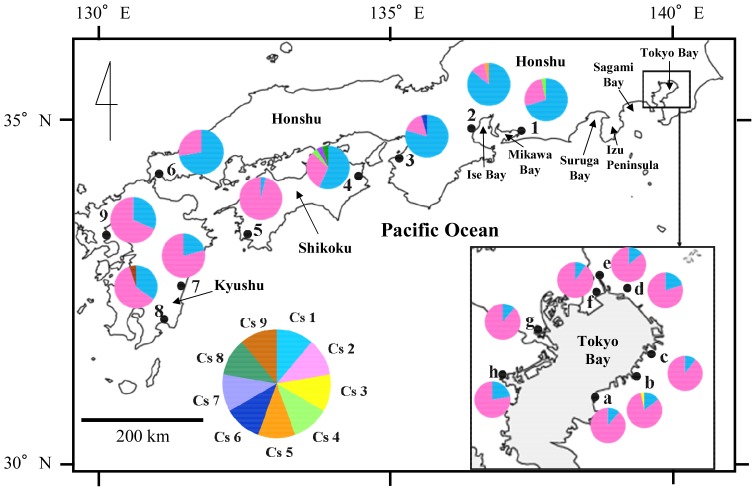
Sampling localities and pie chart representation of mtDNA COI haplotype frequencies for *Clistocoeloma sinense*. See also Tables 1 and 2 for further details.

In 2011 and 2012, sampling of *Clistocoeloma sinense* was conducted by hand-digging on saltmarshes at 8 localities in inner Tokyo Bay and 13 localities on saltmarshes along the western Japanese main Islands from Shizuoka Prefecture to Saga Prefecture, and on Okinawa Island, thereby covering the entire geographical range of *Clistocoeloma sinense* in Japan. In total, 418 samples of *Clistocoeloma sinense* were collected from the 8 Tokyo Bay localities and 9 other localities (Fig. 1, Table 1). No crabs were collected from Izu Peninsula, Okayama, Fukuoka or Okinawa Island. Ethical approval for this research was not required under any local, national or international laws, because all of the animals used in the study were invertebrates. Field collections in two localities included within wildlife protection areas (localities f and g) were authorized by Chiba Prefecture and Tokyo Metropolitan Area local governments. All samples were collected in strict accordance with good animal practice as defined by the relevant national and/or local animal welfare bodies. The third ambulatory leg only was dissected, using the autotomy methods so as to reduce any harmful effects on the sampled individuals, which were then all returned to their places of capture. All pereopod samples were preserved in 99% ethanol immediately after collection.

**Table 1:** Sampling localities and sample size of *Clistocoeloma sinense*.

Locality	Prefecture	Locality number	Latitude	Longitude	n
Obitsu R.	Chiba	a	35° 24′ N	139° 54′ E	28
Shiizu R.	Chiba	b	35° 28′ N	140° 01′ E	28
Tamasaki	Chiba	c	35° 31′ N	140° 03′ E	30
Yatsu tidlflat	Chiba	d	35° 40′ N	140° 00′ E	20
Edogawa R.	Chiba	e	35° 42′ N	139° 55′ E	29
Shinhama lagoon	Chiba	f	35° 40′ N	139° 55′ E	30
Tokyo Port Wild Bird Park	Tokyo	g	35° 34′ N	139° 46′ E	27
Yokohama Port	Kanagawa	h	35° 27′ N	139° 37′ E	18
Kamita R.	Aichi	1	34° 41′ N	137° 19′ E	27
Tanaka R.	Mie	2	34° 47′ N	136° 33′ E	28
Kinokawa R.	Wakayama	3	34° 14′ N	135° 33′ E	24
Katsuura R.	Tokushima	4	34° 02′ N	134° 34′ E	23
Souzu R.	Ehime	5	32° 57′ N	132° 33′ E	28
Koya R.	Yamaguchi	6	34° 03′ N	131° 03′ E	18
Hitotsuse R.	Miyazaki	7	32° 03′ N	131° 29′ E	24
Yoshida R.	Miyazaki	8	31° 27′ N	131° 12′ E	20
Tagori R.	Saga	9	32° 57′ N	130° 12′ E	16

### DNA Sequencing

Total genomic DNA was extracted from the ambulatory leg musculature using a CTAB protocol [11]. The target DNA segment of a portion of the mtDNA cytochrome c oxidase subunit I (COI) was amplified by polymerase chain reaction (PCR) with the primers mtd10 5′-TTGATTTTTTGGTCATCCAGAAGT-3′ [12] and C/N2769 5′-TTAAGTCCTAGAAAATGTTGGGGA-3′ [13]. PCR amplification was conducted in a total volume of 20 µL containing 0.16 µL of TaKaRa ExTaq (5 units/µL), 2.0 µL of 10× Ex Taq buffer, 1.6 µL of dNTP mixture (2.5 mM each); 1.0 µL of each primer (10 mM), and 2.0 µL of template. PCR conditions comprised 35 cycles of denaturation (94°C, 30 s), annealing (45°C, 30 s), and extension (72°C, 60 s) on thermal cyclers in a GeneAmp 9700 PCR System (Applied Biosystems). Amplification products were checked for size by loading 3 µL on a 1.5% agarose gel (GenePure LE) with 0.5 µg/mL ethidium bromide. Subsequent product sequencing was performed at the Dragon Genomics Center [Takara Bio, Otsu, Japan]. Sequencing reactions followed the manufacturer’s suggested protocol. All final sequences were obtained from both strands of gene segments for verification. DNA segments of 528 base pairs of the partial mtDNA COI gene were sequenced and used for DNA analyses. All of the newly obtained sequences were deposited in the DNA Data Bank of Japan (accession numbers AB813717– AB813725).

### Data Analysis

The number of mtDNA COI haplotypes, and the haplotype and nucleotide diversities were calculated for each local population of *Clistocoeloma sinense* by using the computer software program Arlequin Ver. 3.1 [14]. The structure of each population was examined by analysis of molecular variance (AMOVA, [15]), using Arlequin with a model of one group of populations. Pairwise *F*_ST_ (fixation index) values based on uncorrected sequence differences [15], [16] computed using Arlequin were used as indices of genetic differentiation between populations. Significance was calculated for 100,000 permutations using Arlequin and corrected by the false discovery rate method [17]. Population growth evaluations were based on Tajima’s D test [18], [19] and Fu’s Fs statistic [20], using Arlequin. In addition, the fit of the mismatch distribution was compared to that expected under the spatial expansion model with Arlequin. The sum of squared deviations (SSD) and raggedness index (rg) between observed and expected distributions were used as test statistics, the significance being assessed after 1,000 bootstraps. Index of the age of population expansion (τ) was also calculated by Arlequin. To test for evidence of Isolation by Distance, a Mantel test was performed on genetic distance (*F*_ST_/1–*F*_ST_) against geographic distance (minimum coastline distance) between all pairs of sampling localities, using the Isolation by Distance Web Service (Version 3.23) [21]. The significance of Mantel’s Z-test statistic was based on 10,000 permutations.

## Results

**Figure 2:**
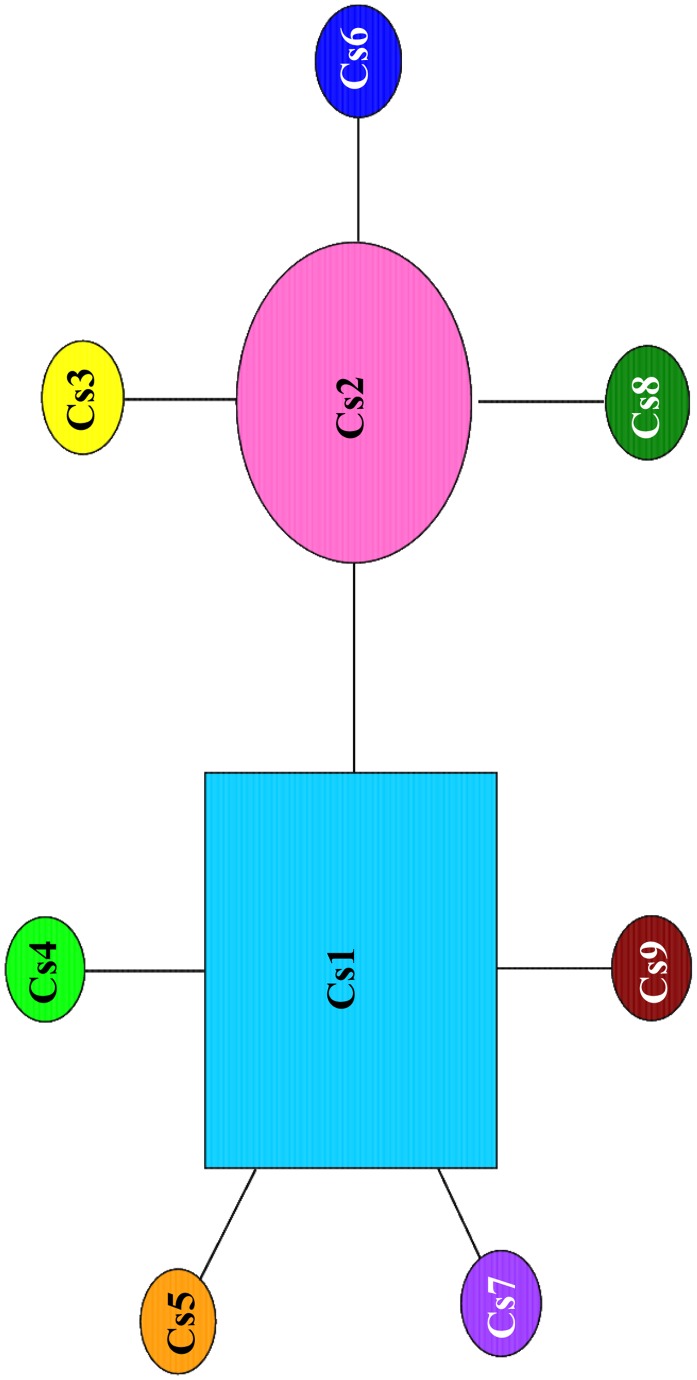
Statistical parsimony network of mtDNA COI haplotypes from *Clistocoeloma sinense* specimens. Each circle and a square represent a unique haplotype. Haplotypes (Cs1–Cs9) correspond to Table 2. Circles and a square size proportional to number of haplotypes.

Nine different mtDNA COI haplotypes, with a total of eight variable sites, were identified from the sequenced samples (*n = *418, Fig. 1, Table 2). No insertions or deletions were found. The nine haplotypes included two that were dominant (Cs1 and Cs2), being found at all of the localities represented. The most frequent haplotype (Cs2) was found at 12 localities (Tokyo Bay, localities a to h; southern Shikoku and Kyushu, localities 5 and 7 to 9), ranging in frequency from 0.60 to 0.96. Haplotype Cs1 was dominant at five localities (numbers 1 to 4 and 6 in central Honshu), ranging in frequency from 0.57 to 0.86. The remaining six haplotypes each occurred only once, at six sampling localities. Haplotype (*h*) and nucleotide (π) diversity values tended to be very low at most localities, with values ranging from 0.0714 to 0.6087 and from 0.000135 to 0.001392, respectively. The Katsuura River population (locality 4) exhibited the greatest genetic diversity (*h = *0.6087 and π = 0.001392), followed by the population from Yoshida River (locality 8) (*h = *0.5421 and π = 0.001146). In contrast, almost all of the Tokyo Bay populations were characterized by low genetic diversity (*h* ranging from 0.1862 to 0.3368; π ranging from 0.000353 to 0.000693). The parsimony network of the mtDNA COI haplotypes of *Clistocoeloma sinense* consisted of two major haplotypes (separated by a single mutational step) and seven minor haplotypes, separated from the major haplotypes by a single mutational step (Fig. 2). Table 2 summarizes the distribution of haplotypes per population.

**Table 2:** Haplotype composition, haplotype diversity (Mean ± SD) and nucleotide diversity (Mean ± SD) of 17 *Clistocoeloma sinense* populations.

Locality number	Haplotype									n	Haplotype diversity	Nucleotide diversity
	Cs1	Cs2	Cs3	Cs4	Cs5	Cs6	Cs7	Cs8	Cs9			
a	3	25								28	0.1984±0.0924	0.000376±0.000537
b	4	23	1							28	0.3148±0.1024	0.000616±0.000713
c	3	27								30	0.1862±0.0881	0.000353±0.000517
d	4	16								20	0.3368±0.1098	0.000638±0.000739
e	4	25								29	0.2463±0.0935	0.000466±0.000606
f	3	27								30	0.1862±0.0881	0.000353±0.000517
g	3	24								27	0.2051±0.0947	0.000389±0.000548
h	4	14								18	0.3660±0.1124	0.000693±0.000781
1	19	7		1						27	0.4530±0.0869	0.000896±0.000895
2	24	3			1					28	0.2619±0.1022	0.000511±0.000640
3	19	4				1				24	0.3587±0.1096	0.000810±0.000845
4	13	7		1			1	1		23	0.6087±0.0818	0.001392±0.001196
5	1	27								28	0.0714±0.0652	0.000135±0.000310
6	13	5								18	0.4248±0.0993	0.000805±0.000854
7	5	19								24	0.3442±0.0987	0.000652±0.000742
8	7	12							1	20	0.5421±0.0763	0.001146±0.001061
9	5	11								16	0.4583±0.0954	0.000868±0.000902

Locality number as given in Fig. 1 and Table 1.

The AMOVA analysis revealed significant overall population differentiation (among populations: % variation = 33.82; within populations: % variation = 66.18, Φ_ST_ = 0.338, *P*<0.001). Pairwise *F*_ST_ values (Table 3) showed a tendency toward genetic differentiation between populations (*P*<0.001 in comparisons of 60 out of all 136 pairs) and had the following pattern: (i) differentiation between Tokyo Bay populations (localities a to h) and central Honshu populations (localities 1 to 4 and 6); (ii) differentiation between central Honshu populations (localities 1 to 4 and 6) and southern Shikoku and Kyushu populations (localities 5 and 7 to 9), excluding the Yoshida River population (locality 8); and (iii) no differentiation between the Tokyo Bay populations (localities a to h) and southern Shikoku and Kyushu populations (localities 5 and 7 to 9), excluding the Yoshida River population (locality 8).

**Table 3:** Pairwise *F*_ST_ values between *Clistocoeloma sinense* populations. Significant probabilities following collection with the FDR method for multiple tests were in bold type.

Locality Number	a	b	c	d	e	f	g	h	1	2	3	4	5	6	7	8
a																
b	−0.027															
c	−0.035	−0.023														
d	−0.009	−0.030	−0.001													
e	−0.032	−0.032	−0.028	−0.029												
f	−0.035	−0.023	−0.034	−0.001	−0.028											
g	−0.038	−0.029	−0.036	−0.013	−0.034	−0.036										
h	0.005	−0.024	0.014	−0.054	−0.021	0.014	0.000									
1	**0.540**	**0.465**	**0.555**	**0.399**	**0.498**	**0.555**	**0.532**	**0.368**								
2	**0.723**	**0.651**	**0.733**	**0.612**	**0.686**	**0.733**	**0.717**	**0.587**	0.032							
3	**0.601**	**0.524**	**0.616**	**0.464**	**0.559**	**0.616**	**0.594**	**0.433**	−0.027	0.000						
4	**0.394**	**0.326**	**0.410**	**0.251**	**0.353**	**0.413**	**0.385**	**0.220**	−0.021	0.088	0.004					
5	0.002	0.028	−0.003	0.096	0.029	−0.003	0.005	0.124	**0.645**	**0.811**	**0.706**	**0.501**				
6	**0.561**	**0.468**	**0.577**	**0.400**	**0.511**	**0.577**	**0.552**	**0.366**	−0.044	0.044	−0.034	−0.033	**0.689**			
7	0.000	−0.023	0.008	−0.048	−0.021	0.008	−0.004	−0.050	**0.394**	**0.601**	**0.458**	**0.252**	0.101	**0.394**		
8	0.165	0.104	**0.181**	0.039	0.123	**0.181**	0.157	0.016	0.155	**0.359**	**0.209**	0.056	**0.296**	0.132	0.038	
9	0.087	0.031	0.102	−0.024	0.045	0.102	0.080	−0.041	**0.257**	**0.488**	**0.322**	0.124	**0.240**	**0.244**	−0.025	−0.039

**Figure 3:**
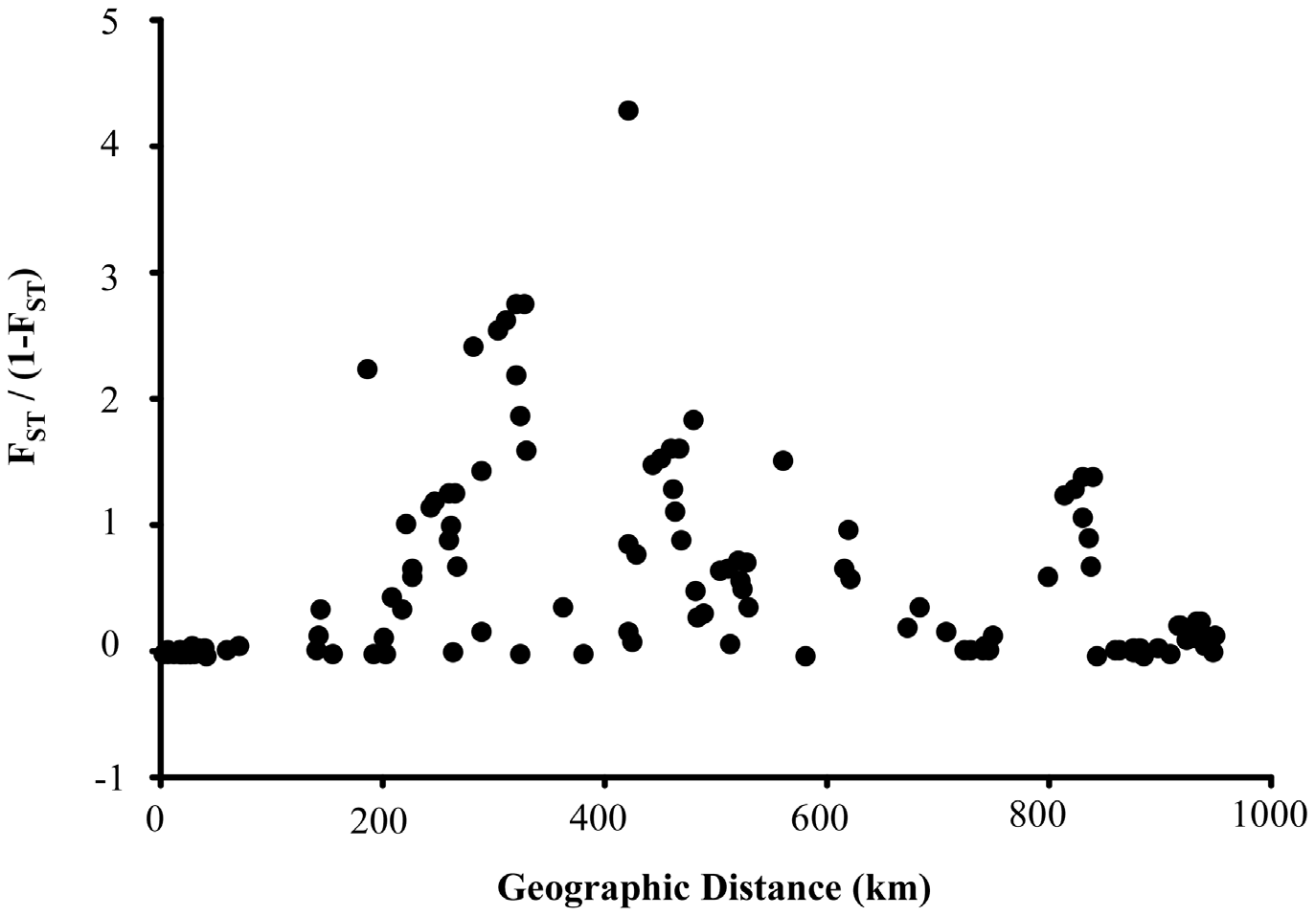
Isolation by distance of *Clistocoeloma sinense* samples. Genetic distances (*F*_ST_/1–*F*_ST_) plotted against geographical distances (minimal coastline distance) among between all localities.

In the demographic analyses (Table 4), Tajima’s D and Fu’s Fs statistics were negative for Tokyo Bay to southern Shikoku population (locality 5), and result of SSD p-value in those populations indicated that each population had undergone a sudden population expansion. Under the assumption of the spatial expansion hypothesis, the value of τ for Tokyo Bay population was lower compared to that of other populations (except for southern Shikoku population). These results show that Tokyo Bay population was critically affected by bottleneck/founder effect in recent years. With respect to Isolation by Distance, no significant relationship between genetic distance and linear geographic distance was apparent within the longitudinal gradient under study (Fig. 3; Mantel Z-test = 30905.65, 10,000 randomizations, *r* = 0.0078, *P* = 0.592).

**Table 4:** Neutrality tests (Tajima’s D) and demographic estimates for mismatch distributions under the spatial expansion model (SSD, rg and τ).

Population	Neutrarity		Demographic		
Locality Number	Tajima’s D	Fu’s Fs	SSD	rg	τ
Tokyo Bay	−0.385	−0.433	**0.002**	0.327	0.287
1	−0.187	−0.114	0.017	0.184	0.606
2	−0.994	−1.128	0.001	0.295	0.315
3	−0.444	−0.382	0.000	0.177	0.460
4	−0.891	−1.742	0.020	0.164	0.880
5	−1.151	−1.155	0.000	0.740	0.172
6	0.870	1.039	0.010	0.203	0.608
7	0.480	0.847	0.003	0.216	0.459
8	0.173	0.153	0.021	0.177	0.765
9	1.034	1.096	0.014	0.217	0.678

SSD: sum of squared deviations between observed and expected distributions; rg: raggedness index; τ: expansion parameter. Significant values after sequential Bonferroni Method are indicated in bold.

## Discussion

### Low Genetic Diversity

All populations of the endangered sesarmid crab *Clistocoeloma sinense* in estuarine saltmarshes located from central Honshu to Kyushu showed low levels of genetic diversity, as measured by haplotype and nucleotide diversities, compared with those of other intertidal brachyuran crabs (Table 5). Grant and Bowen [22] classified marine fishes into four categories on the basis of different combinations of high and low values for haplotype diversity and nucleotide diversity of mtDNA sequences. *Clistocoeloma sinense*, with its low values for both haplotype and nucleotide diversity, is characteristic of the first category, which may have occurred through a recent population bottleneck or founder event involving a single or at most, a limited number of mtDNA linkages.

**Table 5:** Comparison of genetic diversity of mtDNA COI of intertidal brachyuran crabs. Every species was analyzed using the same primers in this study.

		Number				Diversity		
Species	Samplesize	Locality	Haplotypes	Shared haplotypes	Unique haplotypes	Haplotype (h)	Nucleotide (π)	Source
*Scylla serrata*	77	4	24	5	19	0.370–0.850	0.1700–0.4600	[47]
*Chiromantes dehaani*	85	9	25	11	14	0.700–1.000	0.0016–0.0081	[28]
*Neohelice granulata*	69	11	34	3	31	0.454–0.955	0.0013–0.0096	[48]
*Gaetice depressus*	80	8	27	4	23	0.511–0.933	0.0012–0.0042	[28]
*Ptychognathus ishii*	76	6	20	4	16	0.455–0.889	0.0012–0.0024	Kawane et al. unpub.
*Deiratonotus cristatus*	113	13	46	15	31	0.000–0.889	0.0000–0.0230	[29]
*Deiratonotus japonicus*	71	10	34	5	29	0.400–1.000	0.0007–0.0064	[28]
*Deiratonotus kaoriae*	42	2	8	4	4	0.503–0.594	0.0010–0.0056	[49]
*Ilyoplax pusilla*	124	6	40	3	37	–	–	[36]
*Macropthalmus banzai*	124	6	83	30	53	0.673–1.000	0.0019–0.0116	[50]
*Ocypode ceratophthalma*	85	8	26	7	16	0.700–1.000	0.0085–0.0203	[28]
***Clistocoeloma sinense***	**418**	**17**	**9**	**3**	**6**	**0.071–0.609**	**0.00014–0.00139**	**Present study**

Every species was used by the same primers in this study.

The Tokyo Bay populations, located at the northern (and eastern) geographical limits of the species, were characterized by lower genetic diversity than the other local populations. Similar genetic patterns have been previously reported for other marine invertebrate populations representing distributional limits in Japanese waters, viz. the tideland snail *Batillaria zonalis* [23], swimming crab *Portunus trituberculatus* [24], and fiddler crab *Uca arcuata* [25]. Because of the urbanization of coastal areas about Tokyo Bay, about 90% of former tidal flats, including saltmarshes, have been reclaimed, and the remaining limited area habitats isolated [4]. As a result, the population range of saltmarsh-specific species has been reduced, the majority of such populations in Tokyo Bay being now endangered or extinct [4].

### Low Gene Flow

The AMOVA analysis revealed significant overall population differentiation. Differences were apparent by the *F*_ST_ analysis between the (i) Tokyo Bay populations and central Honshu populations and (ii) central Honshu populations and southern Shikoku and Kyushu populations, but not between the Tokyo Bay populations and southern Shikoku and Kyushu populations. According to the Isolation by Distance analysis (Fig. 3), however, there was no significant relationship between genetic distance and linear geographic distance within the longitudinal gradient examined, probably due to the lack of genetic differentiation between the Tokyo Bay and Kyushu populations, despite their broad geographic separation. Removal of Tokyo Bay data from the results indicated that the remaining local populations of *Clistocoeloma sinense* may comprise two genetically deviated groups occurring in the eastern and western parts of the species overall distribution range (Fig. 1). To verify this possibility, IBD was analyzed without Tokyo Bay data. However, no significant relationship between genetic distance and linear geographic distance was apparent within the longitudinal gradient (Figure S1; Mantel Z-test = 7859.62, 10,000 randomizations, *r* = 0.167, *P* = 0.154).

Marine species are generally characterized by metapopulation structures that have a large population size, high dispersal capacity during the pelagic larval stages and extensive distribution [26]. The apparent lack of barriers to dispersal in the marine environment often effectively reduces genetic heterogeneity among populations which makes the differentiation of discreet regional populations difficult [27]. However, some studies have reported that although some intertidal brachyuran crabs have a planktonic larval stage, gene flow between local populations is limited [28], [29]. Similarly, the genetic analysis in the present study revealed genetic differentiation among local populations of Japanese *Clistocoeloma sinense*, indicating limited gene flow between distant local populations, despite their planktonic larval development [30], [31].

The genetic differentiation between geographically distant local populations in Tokyo Bay and Mikawa Bay, demonstrated herein, parallels similar examples of genetic differentiation, also shown by mtDNA analysis, in other marine animal species occurring in Tokyo Bay (including Sagami Bay) and along the coast of western Honshu, viz. the intertidal goby *Chaenogobius annularis* [32], surfperch *Ditrema jordani* [33], and brackish water crab *Deiratonotus cristatus* [29]. Dispersal of these species may be impeded by the extensive land mass of the Izu Peninsula, interrupting gene flow. On the other hand, genetic differentiation between local populations separated by the peninsula has not been found in the intertidal mudskipper *Periophthalmus modestus* [34], Japanese mitten crab *Eriocheir japonica* [35], intertidal rocky shore crab *Gaetice depressus* [28], intertidal mudflat crab *Chiromantes dehaani* [28], or intertidal ocypodid crab *Ilyoplax pusilla* [36]. As indicated by these examples, genetic differentiation patterns can vary between marine species with planktonic larval stages.

Each of the aforementioned intertidal brachyuran crabs have four or more zoea and one megalopa stages [31], [37], whereas *Clistocoeloma sinense* has only three zoea and one megalopa stage [30], [31], suggesting that the latter may have a shorter laraval duration and, therefore, reduced dispersal ability, compared with the other species. However, the larvae of some intertidal brachyuran crabs, even those with four or more zoea stages, remain near their spawning areas, subsequently recruiting to their original or nearby population [38], [39]. Since the larvae of *Clistocoeloma sinense* may have a relatively short planktonic life, it is likely that they remain near their parental population, with little chance of extensive dispersal. This may well have resulted in the genetic differentiation apparent among the regional populations. In the enclosed water body of Tokyo Bay, however, little genetic differentiation was apparent among the discrete local populations, suggesting that strong gene flow occurred between them via larval dispersal. Short distance larval dispersal in an enclosed water body may be adaptive for the maintaining of small local populations as a regional metapopulation.

No local populations of *Clistocoeloma sinense* were found along the coast between Tokyo Bay and Mikawa Bay (a distance of ca. 250 km), despite the existence of small saltmarshes habitat in some estuaries on Shizuoka Prefecture [40]. Some intertidal brachyuran crabs, including *Chiromantes dehaani* and *Eriocheir japonica* are, in contrast, distributed along this stretch of coastline, in Sagami Bay or Suruga Bay [28], [35], which are both characterized by extensive beaches. These crab species are generalists in terms of habitat preference, utilizing various substrate types over wide intertidal range including fresh water zone. By comparison, *Clistocoeloma sinense* is estuarine habitat specialist, utilizing only the saltmarsh habitat in the highest intertidal zone. The discontinuity of local populations of some other habitat specialists, apparently also a consequence of the steep rocky shore lined Izu Peninsula [29], has been observed in other coastal marine animal species, including *Chaenogobius annularis, Ditrema jordani*, and *Deiratonotus cristatus*. Thus, suitable habitat continuity along the coast is important to *Clistocoeloma sinense* for the maintenance of local population networks of marine and brackish water habitat specialists.

The similar genetic structures of the widely separated Tokyo Bay and Kyushu populations of *Clistocoeloma sinense* is suggestive of recent gene flow between the two populations. Unintentional human mediated introduction of the crab to Tokyo Bay from Kyushu is another possibility, as suggested by the genetic analysis of Japanese populations of the venerid bivalve *Phacosoma japonicum* [41]. Sato [41] suggested that juvenile of *Phacosoma japonicum* appear to have been accidentally transported to Tokyo Bay from Ariake Bay (Western Kyushu) together with juvenile short neck clams intended for aquaculture on the tidal flats. Unintended introductions of animal species from foreign countries, including *Phacosoma japonicum* together with short neck clams, have been reported elsewhere, including the tidal flat purse crab *Philya pisum* and other tidal flat animals [42]. These introductions may have occurred when the short neck clams were caught. In the case of *Clistocoeloma sinense*, however, introduction mediated by short neck clam aquaculture is unlikely as these two species live in quite different habitats; the short neck clam lives in sandy sediment on middle to lower tidal flats whereas the crab lives in muddy sediment under higher salt marsh vegetation. Furthermore, aquaculture of transplanted short neck clam began post 1980s in Tokyo Bay [43], whereas Tokyo Bay populations of *Clistocoeloma sinense* have been found in 1952 [44].

Ballast water and ship hull fouling are thought to be major vectors for the introduction of non-indigenous marine animals [45]. In Tokyo Bay, introduced populations of the crabs, *Pyromaia tuberculata, Carcisus estuarii, Phithropanogeus harrisii* and *Acantholoblus pacificus*, following their transportation in ballast water and/or ship hull fouling, have been found since 1980 [46]. These above crabs are subtidal species that can live underwater on ship hulls, a habitat that is not suitable for intertidal crabs, such as *Clistocoeloma sinense.* The possibility of introduction of *Clistocoeloma sinense* in ballast water is also low, since most domestic Japanese ships before the 1950s did not have ballast water tanks. Accordingly, populations of *Clistocoeloma sinense* may have been established naturally before at least 1950, but reasons for the genetic similarity between the Tokyo Bay and Kyushu populations remain unknown. Furthermore, the low genetic diversity and demographic analysis results for these populations suggest that these largely separated local populations may have been established coincidently by the introduction of larvae from unknown another habitat. The haplotype similarity between the Tokyo Bay and Kyushu populations may have resulted from naturally occurring genetic drift. The present data, however, is insufficient for inferring population connectivity. Because microsatellite DNA analysis is likely to be a useful tool for estimating contemporary gene flow among populations, such should be considered in future studies aiming to elucidate the metapopulation structures of *Clistocoeloma sinense*.

### Implications for Conservation of the Tokyo Bay Regional Populations

These findings provide the first substantial genetic information on the endangered sesarmid crab *Clistocoeloma sinense*, such being basic for future conservation and management of local populations of this species in Japanese waters. The Tokyo Bay regional populations of *Clistocoeloma sinense* may warrant special conservation status, as they are already exhibiting relatively low levels of diversity. By way of comparison, the tidal flat snail *Cerithidea cingulata*, formerly with a distribution pattern similar to that of *Clistocoeloma sinense*, occurring in both Tokyo Bay and Mikawa Bay until the 1990s, was not found in Tokyo Bay or along the coastline between the two bays [4]. Planktonic larvae of *Cerithidea cingulata* populations still extant in Mikawa Bay probably cannot disperse to Tokyo Bay due to the lack of suitable interjacent habitat, as in the case of *Clistocoeloma sinense*.

There would appear to be few barriers to the continuation of gene flow between the Tokyo Bay populations of *Clistocoeloma sinense* through larval dispersal. However, the preservation of extant habitats, restoration of saltmarshes and creation of new suitable habitats around the coastline of Tokyo Bay would likely be the most effective measures for conserving this endangered saltmash species.

## Supporting Information

Figure S1 **Isolation by distance of** ***Clistocoeloma sinense*** **samples.** Genetic distances (*F*_ST_/1– *F*_ST_) plotted against geographical distances (minimal coastline distance) without data of Tokyo Bay (Locality Number a∼h).
(TIF)
