# Supplementary figures and images for: Genetic Population Structure of Local Populations of the Endangered Saltmarsh Sesarmid Crab Clistocoeloma sinense in Japan
Source: PLoS One. 2014 Jan 6;9(1):e84720. doi: 10.1371/journal.pone.0084720 (PMC3882244; doi:10.1371/journal.pone.0084720)

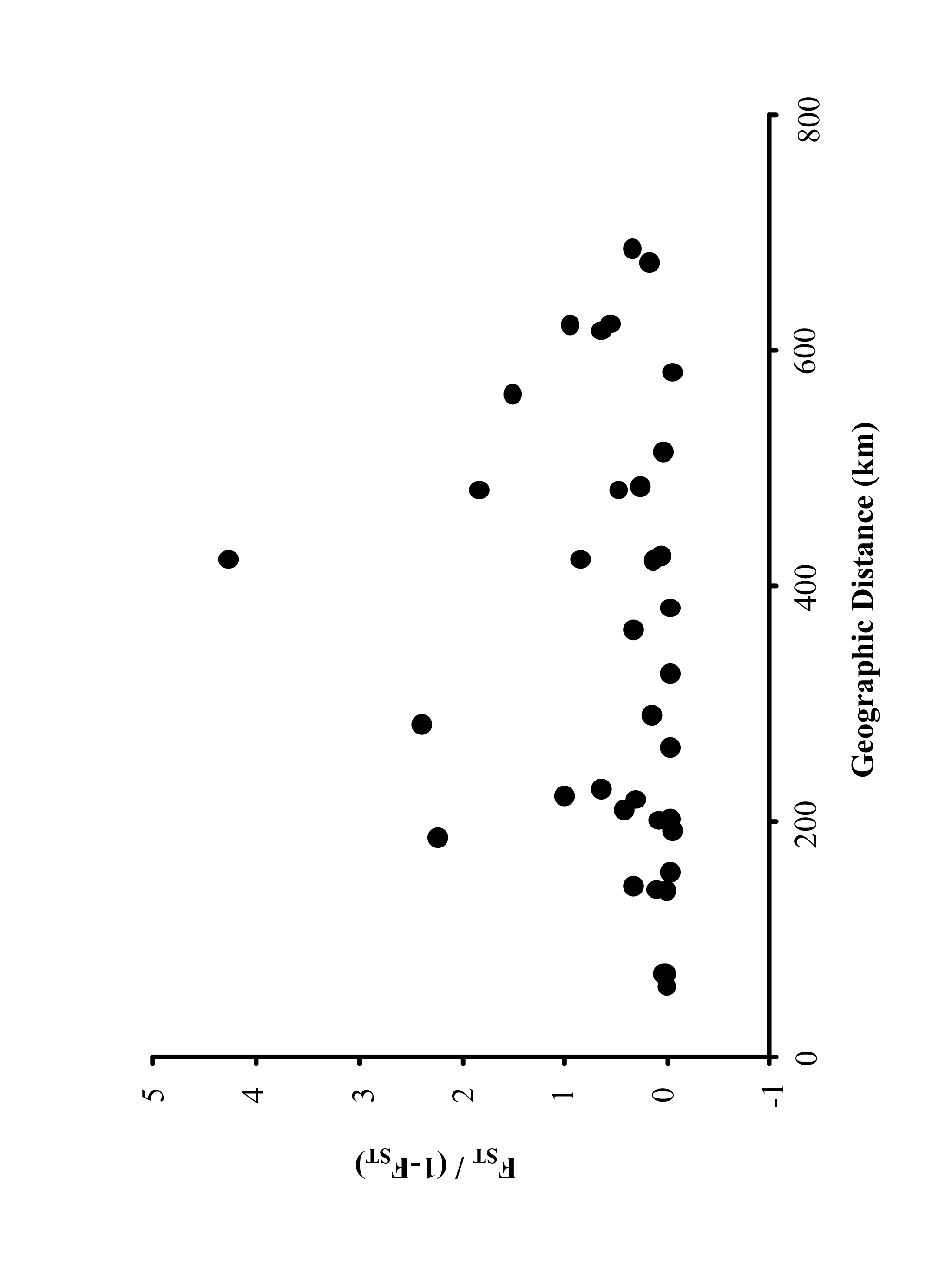

Supplement: Figure S1 — Isolation by distance of Clistocoeloma sinense samples. Genetic distances (F ST/1– F ST) plotted against geographical distances (minimal coastline distance) without data of Tokyo Bay (Locality Number a∼h). (TIF) [file pone.0084720.s001.tif]
